# 

**Published:** 1852-07

**Authors:** 


No. II. JULY. Vol. VIII.

### BUFFALO

### MEDICAL JOURNAL

AND

### MONTHLY REVIEW

OF

### MEDICAL AND SURGICAL SCIENCE.

EDITED BY AUSTIN FLINT, M. D.

BUFFALO:
PUBLISHED BY JEWETT, THOMAS & CO.
Commercial Advertiser Buildings.
. . . . . . . . . . . . . .
1852

*Price* $2.50 *per annum . . . in Advance.*

